# Zirconia Ceramics Doped with Ferrite for Solar Thermal Systems

**DOI:** 10.3390/nano16060346

**Published:** 2026-03-11

**Authors:** Vlad Rada, Mihaela-Ligia Ungureşan, Vasile Rednic, Simona Rada, Florin Lungu, Eugen Culea

**Affiliations:** 1 Management and Economic Engineering Department, Technical University of Cluj-Napoca, 400641 Cluj-Napoca, Romania; v_vlad1944@yahoo.com (V.R.); florin.lungu@mis.utcluj.ro (F.L.); 2 Physics and Chemistry Department, Technical University of Cluj-Napoca, 400641 Cluj-Napoca, Romania; 3 National Institute for Research and Development of Isotopic and Molecular Technologies, 400293 Cluj-Napoca, Romania; vasile.rednic@itim-cj.ro

**Keywords:** ferrite-doped zirconia, solar absorber, solar thermal energy, nanofluid, photothermal conversion, solar thermal efficiency, ferrite

## Abstract

This paper investigates a ceramic material based on ferrite-doped zirconia intended for use as a solar absorber in systems designed for the conversion of solar energy into thermal energy. The experimental study details the synthesis procedure of the ferrite-doped zirconia ceramic and its structural, morphological, optical, and magnetic characterization using X-Ray diffraction (XRD), scanning electron microscopy (SEM), UV–Vis spectroscopy, electron paramagnetic resonance (EPR), and optical band gap energy determination. XRD analysis confirms the presence of the crystalline ferrite phase, which is responsible for the enhanced solar absorption properties. UV–Vis investigations reveal intense absorption bands across the ultraviolet, visible, and near-infrared regions, indicating high solar radiation absorptivity. These properties recommend the investigated ceramic as a promising solar receiver material for solar thermal power plants comparable to conventional materials such as carbides and nitrides.

## 1. Introduction

The growing global demand for energy and the negative environmental impact of fossil fuels necessitate the accelerated development of renewable energy sources. Among these, solar energy represents one of the most promising alternatives due to its inexhaustible nature, wide availability, and low environmental impact. The amount of solar energy reaching the Earth’s surface in a single hour exceeds the annual energy consumption of the entire global population, highlighting the exceptional potential of this resource [1]. However, the main challenge remains the efficient and cost-effective conversion of solar radiation into usable energy. Modern solar technologies include photovoltaic cells, solar thermal systems, and photocatalytic applications. Among these, solar thermal systems are distinguished by their ability to directly convert solar radiation into thermal energy, which can be exploited in residential as well as industrial, medical, desalination, and environmental purification applications. The performance of these systems critically depends on the properties of the solar receiver material, particularly its ability to efficiently absorb solar radiation and to withstand high temperatures.

Solar energy is one of the most promising options of renewable energy available for obtaining a considerable amount of energy, producing electricity and heat without polluting the earth, reducing carbon emissions and replacing fossil fuels.

Solar absorbers are key components of the performance of various solar thermal systems such as thermal power plants and solar thermo-photovoltaics. Theoretical analysis indicates that the ideal solar absorbers are black materials.

The ideal solar absorber would be a single material that absorbs visible light and converts it into heat [2,3,4]. High-tech approaches in this field indicate that the main problem lies in the optimizing of the absorption.

In this context, various metallic and ceramic materials have been investigated for applications as solar receivers. The best performances have been obtained by oxide ceramics, such as alumina, Al_2_O_3_, and zirconia, ZrO_2_, due to their excellent thermal behavior at high temperatures, high oxidation stability and extremely low-cost processing methods [5,6]. Alumina is a white ceramic material with excellent thermal properties and oxidation resistance, but it has a poor absorption of solar radiation, which negatively affects the efficiency of the absorber materials and the entire solar panel system. Alumina (Al_2_O_3_) was extensively studied as a solar receiver material because it offers excellent chemical stability but exhibits low solar absorptance due to its white coloration [7].

Ceramic materials stand out due to their thermal stability, oxidation resistance, durability, and superior reliability at elevated temperatures. Among non-oxide materials, silicon carbide (SiC)- [8] and aluminum nitride (AlN)-based composites [9] have exhibited high solar absorptance; however, their application is limited by poor oxidation resistance, high synthesis costs, and the need for special processing conditions.

In recent years, research efforts have increasingly focused on oxide-based ceramics with enhanced solar absorptance as a viable alternative to non-oxide materials [10]. These materials offer major advantages, including low production costs, high thermal stability, chemical inertness, and superior oxidation resistance. Furthermore, reducing the cost of the solar receiver is essential as it accounts for approximately 20–30% of the total cost of a solar thermal system.

Zirconia (ZrO_2_) is an oxide ceramic of major importance due to its high melting point, thermal stability, oxidation resistance, excellent mechanical properties, and ability to work in severe conditions and it is widely used in a broad range of industrial and technological applications [6]. However, pure zirconia exhibits low solar absorptance, which is primarily limited to the ultraviolet region, thereby restricting its direct use in photothermal applications. They are the absorber materials of the future for the production of energy from concentrated solar radiation with high efficiency and durability. Consequently, the development of zirconia-based ceramics with enhanced solar absorptance represents a current and highly relevant research direction in the field of functional materials for solar energy applications.

Despite the attractive characteristics of these oxides, they do not have the ability to absorb solar radiation and lack sufficient performance to transform it into thermal or electrical energy because of poor optical properties and low thermo-solar efficiency [6]. One of the attempts to reduce the impact these disadvantages of zirconia ceramics is to produce them in black color [2].

On the other hand, varied techniques were used to produce materials used as solar absorbers such as plasma sintering, hot pressing, chemical vapor deposition, self-propagating combustion synthesis and others [11]. These techniques are complicated and require very expensive raw materials. For example, solar absorbers such as SiC, AlN and other composites require high temperature for their sintering (above 2000–2200 °C) and complicated techniques (hot pressing, plasma sintering, and special furnaces operating in an inert atmosphere [12]). In addition, zirconia-based composites have offered promising optical, thermal and mechanical properties compared to SiC- and Al_2_O_3_-based ceramics.

The development of a rapid and efficient method for the synthesis of black solar absorbers has become a necessity in this area. Oxide ceramics are not expensive compared to non-oxide ceramics [10]. In contrast, sintering is a simple and low-cost method. From an industrial point of view, the sintering and sol–gel processes are suitable and preferred as preparation methods of advanced materials with high efficiency and low cost.

The high costs and the complicated processing methods of carbide- and nitride-based absorber materials recommend black zirconia as a promising material for high-temperature solar receivers with high efficiency and durability [13].

The main problems of black zirconia ceramics prepared by different synthesis methods are that (i) the phase structure of black ceramics is disordered such that it leads to drastic changes in volume, cracks, and involves the reduction of mechanical properties; and (ii) instability at high temperature and poor color saturation [14].

Zirconia has three polymorphic forms: monoclinic, tetragonal and cubic crystalline phases. At higher temperatures (>1150 °C) zirconia undergoes a tetragonal to monoclinic transformation which is characterized by volume expansion (4%) and the formation of microcracks in zirconia compacts upon cooling. By incorporating of stabilizing dopants such as Y_2_O_3_, CeO_2_, MgO, and CaO, the tetragonal and cubic polymorphs in zirconia are retained upon cooling and the microcrackings are reduced. Zirconia stabilized with 15–24% MgO is comparatively cheaper compared with other oxides; for example, ZrO_2_–Y_2_O_3_ (with 7–8% Y_2_O_3_). The fabrication of crack-free zirconia compacts with in situ MgO doping reveals that MgO nanoparticles cause a reduction of the stress and cracking [15].

Recent studies have demonstrated that doping zirconia with metal oxides such as Fe_2_O_3_ and MnO_2_ leads to the formation of black ceramics with high solar absorptance, achieved through simple and cost-effective processing methods. Achieving bulk blackening of zirconia not only enhances the efficiency of solar radiation absorption but also improves its thermal and mechanical properties, which are essential for applications in solar thermal systems.

Functional analysis reveals that the effectiveness of Fe_2_O_3_ is determined not only by band gap or electron affinity but also by adaptability to the device, charge transport and phase stability [16,17].

In this study, iron trioxide was selected for the blackening of zirconia because it can produce ferrite in the host matrix. In this context, the present study aims to develop a black zirconia material having the 40Fe_2_O_3_·60[ZrO_2_·MgO] composition in the mole % with advanced crystallinity and desired phases that provide the color intensity required in solar thermal applications. The preparation of ferrite-doped zirconia was realized by solid-state reactions at high temperatures. The structural and optical characterization of prepared materials were realized by X-Ray diffraction (XRD), micrographs SEM, ultraviolet–visible (UV–Vis) and electron paramagnetic resonance (EPR) spectroscopy.

## 2. Experimental Procedure

The solar absorber was obtained in the form of a ZrO_2_–MgO-based ceramic doped with 40% Fe_2_O_3_. For comparative purposes, an undoped ZrO_2_–MgO reference ceramic was also prepared. Both ceramic systems, ZrO_2_–MgO–Fe_2_O_3_ and ZrO_2_–MgO, were synthesized using the high-temperature solid-state reaction method, employing high-purity analytical-grade metal oxides (Sigma–Aldrich, St. Loius, MO, USA) as the starting materials.

**Figure 1:**
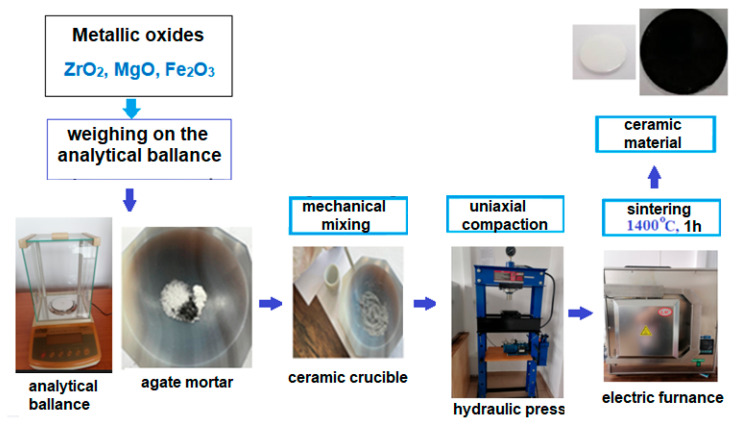
The chart flow of the preparation of ceramic products.

For the synthesis of the ZrO_2_–MgO ceramic doped with 40 mole % Fe_2_O_3_, appropriate amounts of powders were accurately weighed using an analytical balance: 0.4563 g ZrO_2_, 0.1492 g MgO, and 0.3945 g Fe_2_O_3_. The powders were homogenized by fine grinding in an agate mortar, followed by additional milling in a ball mill. The resulting mixture was uniaxially pressed into pellets with a thickness of approximately 2 mm and a diameter of 9 mm using a 50 t hydraulic press. The pellets were placed in an alumina crucible and sintered in an electric furnace at 1400 °C for a dwell time of 1 h. After sintering, the samples were rapidly cooled by removing the crucible from the furnace and transferring the pellets onto a stainless-steel plate at room temperature. In order to prepare the ZrO_2_–MgO reference ceramic, 0.7335 g ZrO_2_ and 0.2465 g MgO were used. Figure 1 shows a typical process flow chart outlining all the steps along with the starting process time.

After sintering, a clear color change was observed, from white for the undoped ceramic to black for the Fe_2_O_3_-doped samples, indicating the formation of absorbing phases responsible for the enhanced solar absorptance [18].

The crystalline or amorphous characteristics of the synthesized samples were investigated using a Rigaku X ray diffractometer (Hong Kong, China) equipped with a graphite monochromator and a copper anode tube (λ = 1.54 Å). UV–VIS spectra were recorded with a UV–VIS spectrometer of Perkin–Elmer Lambda 45 type (Waltham, MA, USA), equipped with an integrating sphere and an accuracy of the wavelength of ±2 nm. Electron paramagnetic resonance (EPR) measurements were conducted in the X-band frequency range (9.52 GHz) using a Bruker ELEXSYS 500 spectrometer (Billerica, MA, USA).

## 3. Results and Discussions

### 3.1. The Investigation of the Structural Properties of Ceramic Materials by X-Ray Diffraction (Xrd)

**Figure 2:**
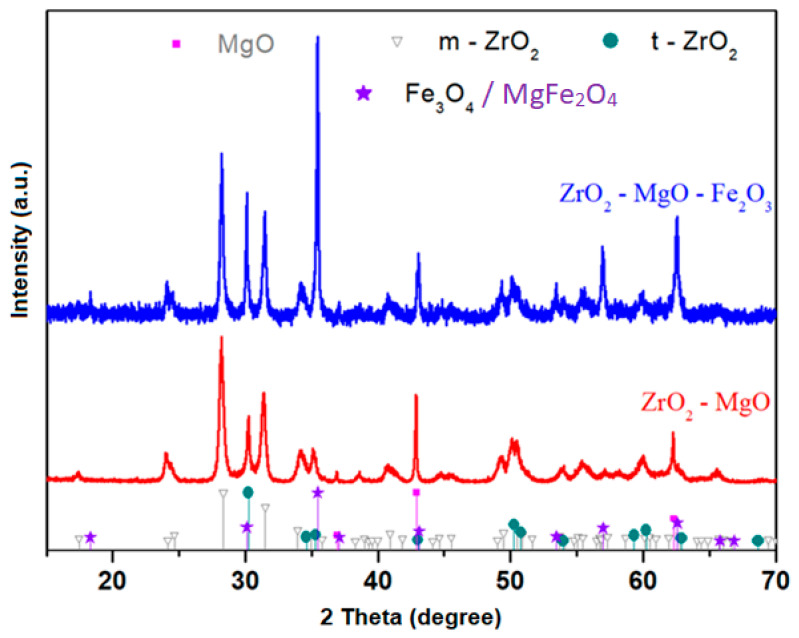
X-Ray diffraction (XRD) patterns of the prepared ceramic samples. All peaks of the Fe_3_O_4_ crystalline phase are overlapped with the diffraction peaks of the MgFe_2_O_4_ crystalline phase.

The X-ray diffraction patterns of the obtained ceramic samples are presented in Figure 2. XRD analysis reveals the presence of the following crystalline phases in the zirconia-based ceramic structure: ZrO_2_ with monoclinic and tetragonal structures, MgO with a cubic structure, and the MgFe_2_O_4_ (ferrite) phase with a cubic structure. The introduction of high iron oxide contents, up to 40 mol%, into the ZrO_2_–MgO ceramic matrix leads to a progressive reduction of the crystalline MgO phase and the emergence of the MgFe_2_O_4_ crystalline phase. The magnesium ferrite MgFe_2_O_4_ crystalline phase have overlaps in XRD with the Fe_3_O_4_ crystalline phase (see Figure S1). The presence of the ferrite phase accounts for the chromatic transition of the ceramic from white to black.

The parameter of the unit cell, (*a*, *b*, *c*), proportion of phases and the effective crystallite mean size, *D_eff_* (nm), were determined using a Rietveld refinement which provide a more reliable evaluation of crystallite size and phase fractions. The crystal structure parameters obtained by Warren-Averbach Fourier analysis by using the POWDER CELL 2.3 computational program are listed in Table 1.

Table 1 also presents the values of the average particle size for all crystalline phases of the prepared material considering both individual ferrite phases. For the iron sample, the average particle size ranges from 27.01 to 80.87 nm. The particle size of the ferrite crystalline phase is responsible of photothermal conversion. These values indicate that the ferrite phase consists of nanometer-sized crystallites which contribute to the optical and magnetic properties of ceramic material. Consequently, a ceramic absorber with average particle sizes below 100 nm was obtained, a feature that is important for enhancing solar absorption properties.

The contents of monoclinic zirconia, tetragonal zirconia and MgFe_2_O_4_ phases were found to be 65%, 6.8% and 28.2%, respectively.

The volume fraction of monoclinic zirconia, *R*, can also be estimated using the calculation of the intensity ratio described from the quantitative analysis of the monoclinic and tetragonal zirconia system by XRD [19]. For the monoclinic, the used XRD peaks for (111) planes are located at 28.2° and 31.5°, while for tetragonal phase, the peak is labeled at 30.2°.

$$R=\frac{I_{m\left(28.2^{0}\right)+}I_{m\left(31.5^{0}\right)}}{I_{m\left(28.2^{0}\right)+}I_{m\left(31.5^{0}\right)+}I_{m\left(30.2^{0}\right)}}$$

Volume fraction, *R*, for monoclinic zirconia is estimated at 68.55%.

**Figure 3:**
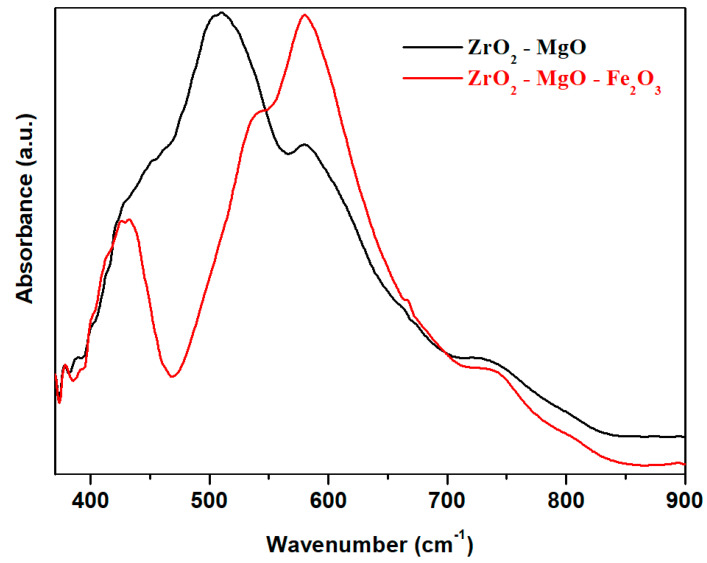
FTIR spectra of the zirconia ceramics.

Spinels have two main metal–oxygen vibrational bands in IR spectra and they can be differentiated by these. The Fe_3_O_4_ crystalline phase shows the higher frequency A-site vibrations of around 570–580 cm^−1^ and lower frequency B-site vibrations of around 380–400 cm^−1^. For the MgFe_2_O_4_ phase, the Mg^2+^ on A-site shifts the A-site IR band downward to 540–560 cm^−1^ while B-site IR band can shift upward slightly to 410–430 cm^−1^. For our sample, three individual IR bands centered at about 425, 432 and 540 cm^−1^ appear comparatively with host matrix (see Figure 3) which can be assigned with the formation of the MgFe_2_O_4_ crystalline phase. The intensity of the IR bands situated between 380 and 400 cm^−1^ do not change by doping with iron (III) oxide. The intensity of IR bands centered at about 580 cm^−1^ was increased by the addition of iron (III) oxide. The results can be associated with the presence of a mixed ferrite phase in the zirconia matrix.

At 1400 degrees under air atmosphere, the stabilization of magnetite (Fe_3_O_4_) is thermodynamically unfavorable, whereas the formation of MgFe_2_O_4_ in the presence of MgO is expected. A detailed study of both crystalline phases is discussed in the Supplementary Information (see Table S1).

Numerous studies have demonstrated that the suspension of solid particles in fluids leads to an increase in their thermal conductivity, which in turn enhances heat transfer efficiency [20,21]. In the past, fluids containing micron-sized or larger particles exhibited significant drawbacks, such as particle sedimentation, channel blockage, and equipment wear [4,22]. To overcome these limitations, the use of nanoparticles was proposed, giving rise to a new class of fluids with enhanced thermal conductivity. With the rapid development of nanotechnology, nanoparticles with sizes below 100 nm have been produced, enabling the formation of stable suspensions and the improvement of the thermal properties of the base fluid [23,24]. Furthermore, it has been shown that the addition of small concentrations of metallic or metal oxide nanoparticles to a fluid can significantly increase its thermal conductivity.

In conclusion, magnesium oxide enters in the form of a solid solution and can contribute to the host structure with a dual role as a stabilizer of monoclinic zirconia and as a ferrite former. The prepared absorber material consisting of nanoparticles smaller than 100 nm can be recommended for use as a nanofluid in photothermal energy conversion systems.

### 3.2. Infrared (Ir) Spectra

Infrared spectra of the prepared samples are shown in Figure 3. In the ZrO_2_–MgO ceramics, the broadening IR bands located in the region between 360 and 750 cm^−1^ are attributed to the presence of the ZrO_2_ crystalline phases with monoclinic and tetragonal structures (~600 cm^−1^) [25]. The IR band located at about 520 cm^−1^ can be correlated to the stretching vibrations of Mg–O bonds and the bending vibrations of O–Mg–O angles, and overlap with the stretching vibrations of Fe–O bonds. The IR bands located at about 435 and 580 cm^−1^ correspond to the Fe–O vibrations from [FeO_6_] and [FeO_4_] structural units.

By adding iron trioxide in the host matrix, typical IR bands of ferrite and zirconia appear. The first IR bands situated in the range between 350 and 470 cm^−1^ show some splitting due to the ferrite crystalline phase. In ferrite, the characteristic absorption bands of the tetrahedral complexes are higher than that of the corresponding octahedral modes. The IR band evidenced at around 410 cm^−1^ corresponds to the octahedral of ferrite and the split into sub-bands observed at 425 and 435 cm^−1^ are assigned to the tetrahedral band position. The absorption bands assigned to tetrahedral metal ions can be seen at 535, 665 and 725 cm^−1^. The presence of Fe^2+^ ions in ferrites can produce the splitting of the absorption bands or can cause new shoulders. These structural evolutions can be due to the Jahn Teller distortion caused by Fe^2+^ ions and local deformations in the crystal fields.

The second region of IR bands centered at about 540 and 580 cm^−1^ are associated with stretching vibrations of Zr–O bonds in the monoclinic and tetragonal ZrO_2_ crystalline phases. The intensity of the last band increases by doping with Fe_2_O_3_, suggesting the improvement of the host matrix with the tetragonal ZrO_2_ phase in agreement with the XRD analysis. This evolution can be explained considering that MgO plays the role of a stabilizer of zirconia.

The covalency of the Fe–O bond is stronger than that of the Zr–O or Mg–O bond because the values of the electronegativities for Fe, Zr, Mg and O are 1.8, 1.4, 1.2 and 3.5. By adding an excess of oxygen in the host matrix, iron has a higher affinity to attract oxygen atoms and to produce [FeO_4_] and [FeO_6_] structural units than that of its analogs. The accommodation of the lattice with oxygen atoms occurs through the formation of higher-coordination iron which results in the appearance of the ferrite crystalline phase. Further, the excess of oxygen will be responsible for the stabilization of the tetragonal zirconia phase. These mechanisms explain the partial conversion of the monoclinic into the tetragonal zirconia phase.

In conclusion, the magnesium–zirconia system is converted in iron–magnesium–zirconia systems by doping with higher Fe_2_O_3_ content. The addition of an excess of oxygen in the host matrix implies two mechanisms: (i) the formation of octahedral and tetrahedral [FeO_n_] structural units due to the higher affinity of iron towards oxygen which produce the ferrite crystalline phase; and (ii) the increase in fractions of the tetragonal ZrO_2_ crystalline phase by the diminishing of the monoclinic zirconia phase. The system behaves as a magnesium–zirconia glass ceramic enriched with ferrite.

### 3.3. Characterization of the Optical Properties of Zirconia Ceramics

**Figure 4:**
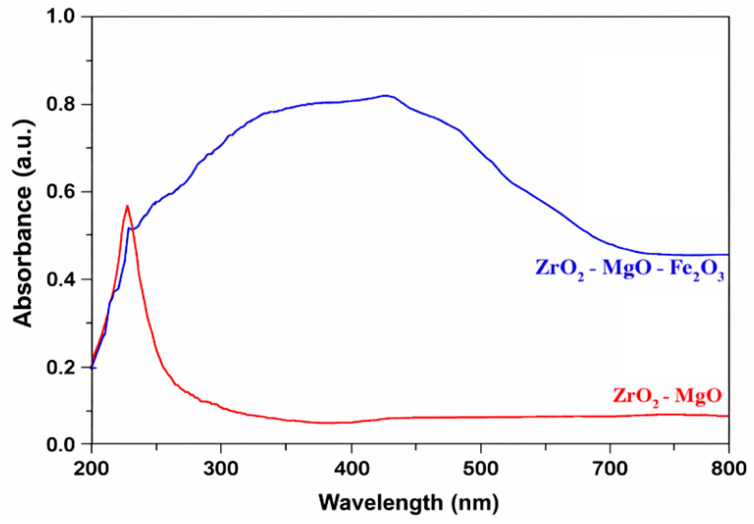
UV–Vis spectra of undoped ZrO_2_–MgO ceramics and ZrO_2_–MgO ceramics doped with 40% Fe_2_O_3_.

**Figure 5:**
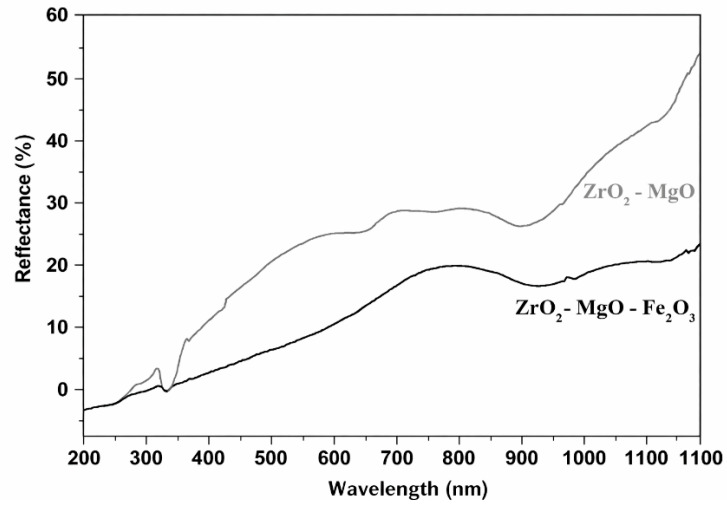
UV–Vis reflectance spectrum of the prepared ceramics.

Figure 4 and Figure 5 show the Uv–Vis absorption and reflectance spectra of the prepared ceramic products.

From the UV–Vis absorption spectrum recorded in the 300–800 nm range for the ZrO_2_–MgO ceramic, a distinct absorption band is observed, with a maximum located at 235 nm in the ultraviolet region. This UV–Vis band is attributed to O^2−^ → Zr^4+^ charge-transfer transitions in zirconia [26]. Zirconia absorbs exclusively in the ultraviolet region, which makes it inefficient for harvesting solar radiation in the visible range [27]. In this region, a maximum optical absorption of approximately 56% is obtained, with no significant absorption bands observed in the visible domain.

The addition of Fe_2_O_3_ to the zirconia matrix significantly modifies the UV–Vis absorption characteristics of the ceramic. As a result, broader absorption bands appear in the 250–800 nm range. The UV–Vis band centered at approximately 388 nm, with an optical absorbance of 77%, is attributed to isolated Fe^3+^ ions located in sites with an octahedral coordination. The intense UV–Vis band at 460 nm corresponds to electronic transitions of Fe^3+^ ions located in distorted octahedral geometries, while the band in the 585–785 nm range originates from electronic transitions of Fe^3+^ ions arranged in both octahedral and tetrahedral geometries with oxygen ions [28]. The strong and extended absorption in the ultraviolet and visible regions is attributed to direct O^2−^ → Fe^3+^ charge-transfer transitions in the UV region and to 3d → 3d electronic transitions of Fe^3+^ ions in the visible region.

Fe^3+^ ions located in octahedral geometries involve t_2g_ → e_g_ electronic transitions, generating UV–Vis bands in the 850–1100 nm range. The increase in the intensity of UV–Vis bands in the 300–1100 nm range can be correlated with the presence of both Fe^2+^ and Fe^3+^ ions, as well as with the formation of the MgFe_2_O_4_ ferrite crystalline phase.

Consequently, the addition of Fe_2_O_3_ leads to a maximum optical absorption of approximately 80% in the visible light region, highlighting the beneficial role of iron (III) oxide in enhancing the optical properties of the black ceramic [29].

**Figure 6:**
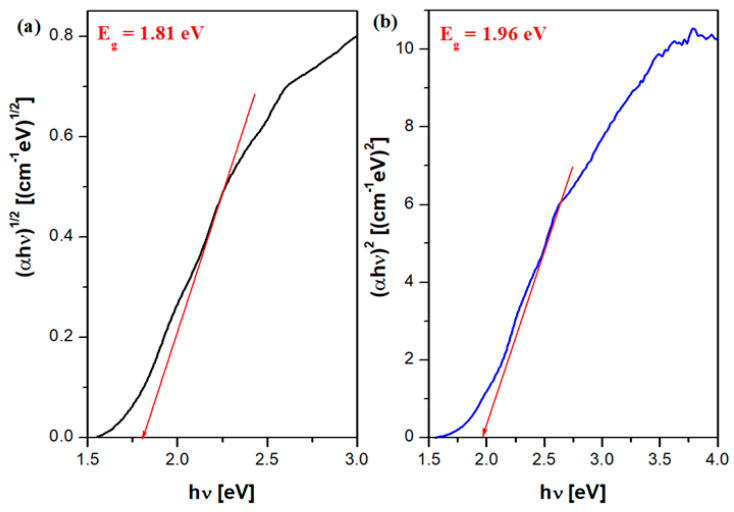
Graphical representation of (**a**) (*αhν*)^1/2^ versus hν and (**b**) (*αhν*)^2^ versus *hν* for the ZrO_2_–MgO–Fe_2_O_3_ ceramic. The red line represents the extrapolation used to determine the optical band gap energy, *E_g_*.

A similar trend of improved solar light absorption for iron-containing ceramics can also be observed from the diffuse reflectance spectra shown in Figure 6.

The Fe_2_O_3_-based ceramic exhibited lower diffuse reflectance values over the entire 200–1100 nm range compared to the undoped sample. In the visible region, the ferrite-doped ceramic showed a maximum reflectance of 21.27%, compared to 28.97% for the undoped ceramic. In the ultraviolet region, the maximum diffuse reflectance values were 3.59% and 6.36% for the doped and undoped samples, respectively. In the infrared region (at 1100 nm), the maximum reflectance values were 24.24% for the doped ceramic and 51.37% for the undoped ceramic.

These results indicate that the addition of iron (III) oxide significantly enhances solar light absorption in the visible and infrared regions compared to the undoped ceramic, which absorbs efficiently only in the ultraviolet region. Consequently, ferrite-doped ceramics can be considered efficient solar absorber materials with high potential for solar energy harvesting applications.

In the literature data, the black ZrO_2_/Fe_2_O_3_/MnO_2_ composites with 10–30 wt % Fe_2_O_3_ were prepared by the sintering method at a temperature of 1700 °C for 2 h [10]. The ZrO_2_/MgO/Fe_2_O_3_ ceramics with 40 mole% Fe_2_O_3_ are produced by high temperature solid-state reactions which combine the sintering method with the melt quenching method at a temperature of 1400 °C for 1 h. Our sample was produced at high efficiency with low processing costs.

### 3.4. Estimation of the Optical Band Gap Energy of the Prepared Material

The optical band gap energy (*E_g_*) values can be determined from plots of (*αhν*)*^n^* versus *hν*, where *n* = 1/2 corresponds to indirect transitions and *n* = 2 corresponds to direct transitions [27]. The corresponding plots for the ferrite-based ceramic, (*αhν*)*^1/2^* or (*αhν*)*^2^* versus *hν*, are shown in Figure 6. Both direct and indirect band gaps for multiphase ceramic systems are generally determined by the present of the individual phases or by the modifications in composition (for example, in doping).

The values of *E_g_* were estimated from the extrapolation of the tangent fit of these curves corresponding to the edge of fundamental absorption. The error of the estimated *E_g_* values can be between 0.5 and 1 eV [30].

The optical band gap energy of the ferrite-doped ceramic was determined to be *E_g_* = 1.81 eV for indirect transitions and *E_g_* = 1.96 eV for direct transitions. These values indicate a semiconducting behavior [31], as the optical band gap energy is lower than 3 eV, a characteristic that is relevant for photothermal applications.

For the ZrO_2_–MgO ceramic, the optical band gap energy values were determined to be 4.1 eV (for *n* = 1/2) and 4.7 eV (for *n* = 2). The significant reduction in the band gap energy to 1.81 eV (indirect transitions) and 1.96 eV (direct transitions) in the case of the iron-ion-doped ceramic can be attributed to increased octahedral distortions and Fe–O rearrangements of octahedral molecular orbitals.

These band gap modifications also contribute to the enhanced solar light absorption observed for the iron-containing ceramic.

### 3.5. Photoluminescence (Pl) Spectra of the Studied Samples

**Figure 7:**
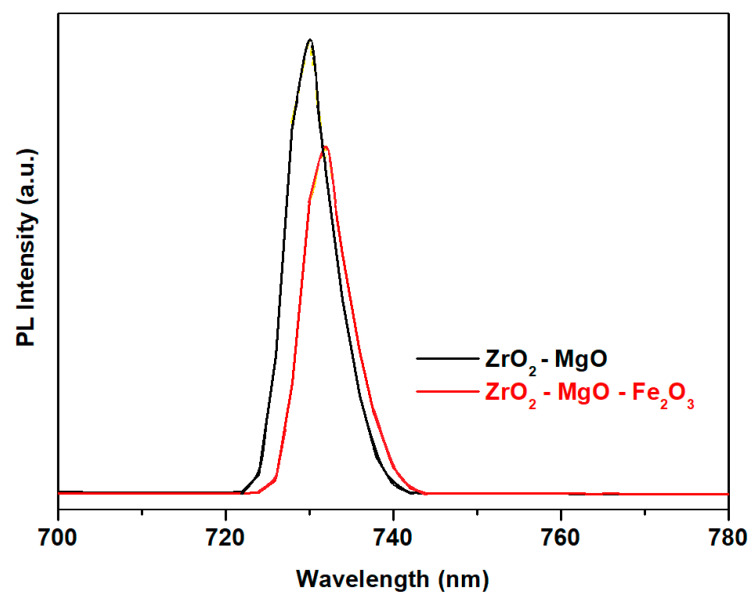
PL spectra of the prepared samples.

The PL spectra recorded for undoped and doped zirconia ceramics are indicated in Figure 7. The PL spectra reveal distinct emission characteristics which reflect the influence of Fe_2_O_3_ content on lattice modifications and the defect chemistry on ferrite structure. An inspection of photoluminescence spectra of the undoped and doped zirconia ceramics suggests an abrupt decrease by adding Fe_2_O_3_ content in the host matrix. This evolution is expected due to the change in the crystallite sizes in agreement with XRD data or/and the accumulation of oxygen vacancy numbers.

The shape of the emission peaks seem to have the same similarities for both samples. The highest emission peak situated at 730 nm is caused by oxygen interstitials. The presence of iron atoms in this sample causes smaller photoluminescence and a decrease in luminescence intensity in the ZrO_2_–MgO–Fe_2_O_3_ sample. The position of the PL band shifts to the near-infrared wavelength region situated at 732 nm for the doped samples. This evolution leads to the conclusion that the photoluminescence mechanism in iron–zirconia ceramic is different from that of zirconia ceramic. The photoluminescence intensity in the near-infrared region is more dominated because it is associated with radioactive recombination.

In conclusion, by doping with iron trioxide, the decline in luminescence can be due to the formation of secondary phase (ferrite crystalline phases) and pronounced microstructural changes.

### 3.6. Structural Characterization by Electron Paramagnetic Resonance (Epr) Spectroscopy

Iron ions can exist in ceramics in the +2 or +3 oxidation states. However, only Fe^3+^ ions generate EPR signals due to their 3d^5^ electronic configuration [32]. Fe^3+^ ions possess unpaired electrons in their 3d orbital configuration and thus exhibit paramagnetic properties.

**Figure 8:**
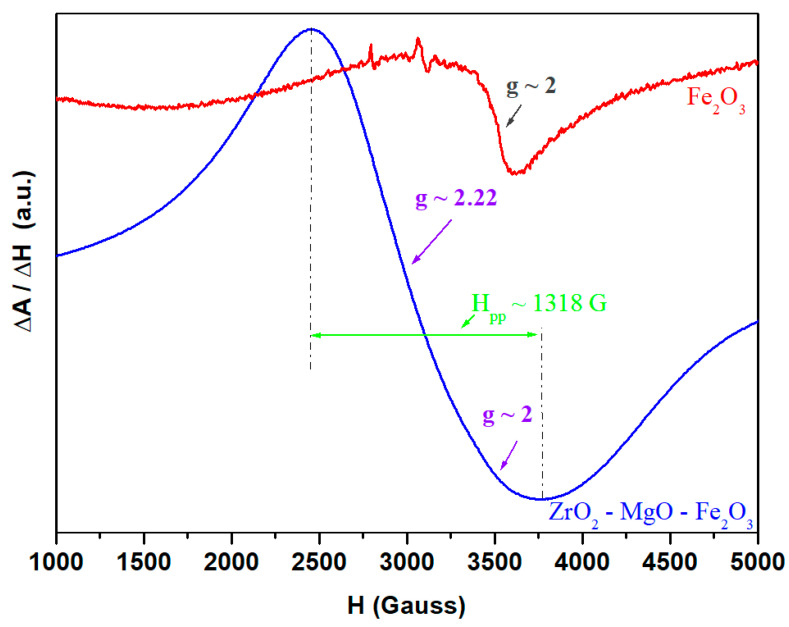
EPR spectra of iron(III) oxide used as a raw material and of iron-based ceramics.

The EPR spectra of iron (III) oxide used as a raw material and of iron-based zirconia ceramics are presented in Figure 8.

In the case of iron (III) oxide powder, a resonance line centered at a gyromagnetic factor, *g*~2, is observed, which is characteristic of isolated Fe^3+^ ions arranged in octahedral geometries with a weak crystal field or presented as clustered entities [33]. The narrower signal at *g*~2 indicates weakly interacting Fe^+3^ ions. The dramatic broadening of the signal in the iron-based ceramic shows the formation of spinel ferrite, ferromagnetic ordering and strong superexchange interactions.

For the ZrO_2_–MgO–Fe_2_O_3_-based ceramic, a broad and strong resonance signal centered at *g*~2.22 was detected. Line width and value of gyromagnetic factor, *g*, are influenced by magnetic dipolar and superexchange interactions. This signal is assigned to the clustered Fe^3+^ ions too.

Strong dipolar interactions give large values of gyromagnetic factor, *g*, and peak-to-peak line width (*H_pp_*), whereas strong superexchange interactions give smaller values of *g* and *H_pp_*. The increase in the value of the *g* and *H_pp_* are, respectively, *g*~2.22 and *H_pp_* = 1318 G for iron–zirconia ceramic when compared with Fe_2_O_3_ powder results in the improvement of dipol–dipol interactions. The shape and position of these absorption features suggest that in the case of the studied samples, the dipole–dipole interactions are predominant relative to superexchange interactions.

The EPR spectrum clearly differentiates the MgFe_2_O_4_ crystalline phase from the Fe_3_O_4_ crystalline phase. It shows a ferrimagnetic-type line with a broad signal, with *g*~2.0–2.22 and a peak-to-peak line width of *H_pp_*~1318 G, is consistent with MgFe_2_O_4_ spinel formation, and shows no clear evidence of Fe_3_O_4_ mixed valence behavior. Furthermore, the line width of ~1318 G is very typical for MgFe_2_O_4_ nanoparticles. If Fe_3_O_4_ were dominant, a stronger asymmetry of a more collapsed broad line with intense superexchange interactions should be observed. That evolution favors the MgFe_2_O_4_ crystalline phase rather than the Fe_3_O_4_ crystalline phase.

In conclusion, zirconia–iron (III) oxide-based ceramics can be identified as a more cost-efficient alternative to other black absorbing materials, which typically require sintering temperatures above 2000 °C and highly demanding processing conditions. Iron-doped zirconia systems allow densification at significantly lower temperatures (approximately 1400 °C) using conventional air-fired furnaces, thereby reducing both energy consumption and technological complexity.

### 3.7. Structural Analysis by Scanning Electron Microscopy (SEM)

Based on the obtained results, the ceramic material prepared for photothermal system applications exhibits the following characteristics:

- An average particle size below 90 nm.
- An optical band gap energy, $E_{g}<3\;\mathrm{eV}$, that confers semiconducting properties.
- An optical absorbance of approximately 80% in the visible light region.
- A diffuse reflectance of about 20% in the visible region.

The developed product shows selectivity dependent on the type of ions present and indicates high efficiency as a solar absorber.

**Figure 9:**
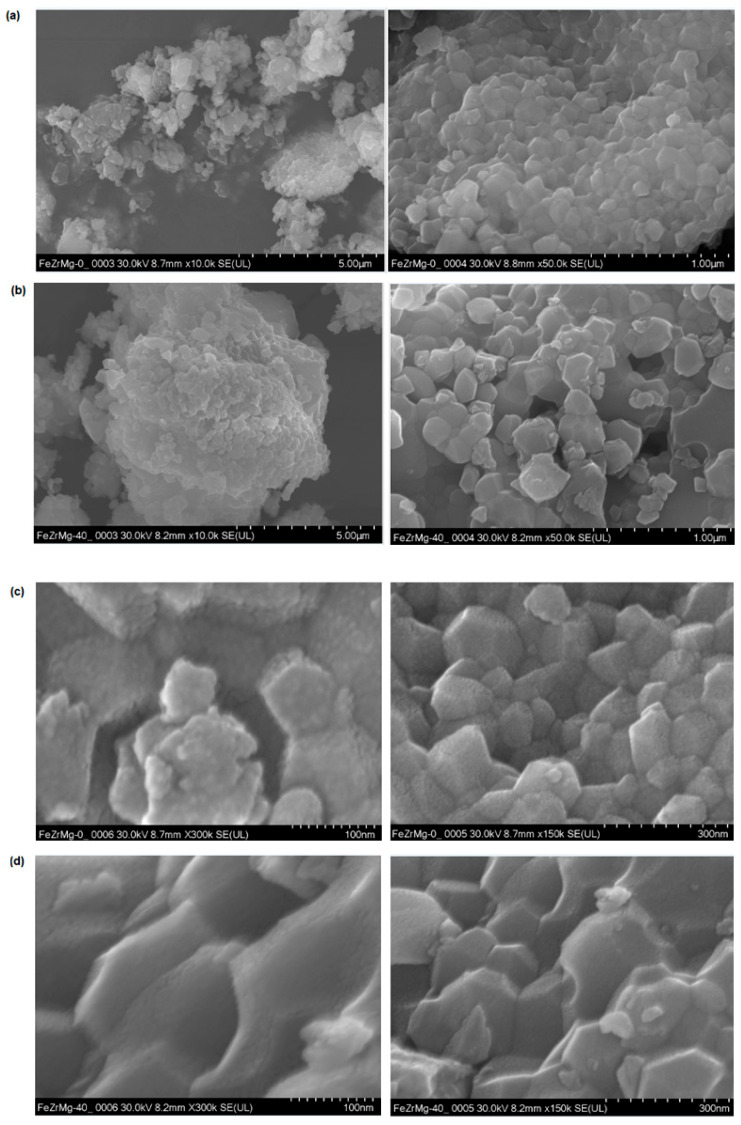

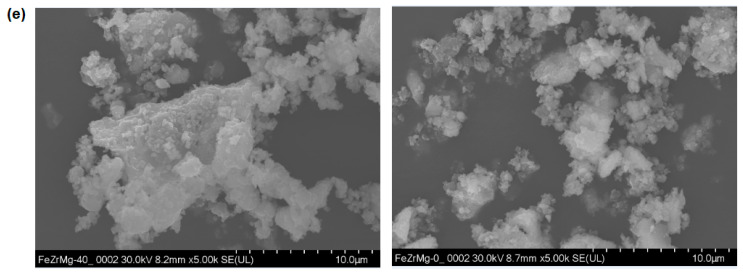
SEM micrographs of the (**a**), (**c**) ZrO_2_–MgO, (**b**), (**d**) ZrO_2_–MgO–Fe_2_O_3_, and (**e**) ZrO_2_–MgO–Fe_2_O_3_ and ZrO_2_–MgO samples at 10 µm.

Figure 9 shows the SEM images of the structure of the prepared materials. The ZrO_2_-based ceramic was obtained via a solid-state reaction route at 1400 °C and is available in the form of disks with a diameter of 9 mm and a thickness of 2 mm. The SEM images show agglomerated grains with homogeneous regions and well-defined crystallites, with sizes below 100 nm, in agreement with the data obtained from XRD analysis.

The SEM images at high and low magnification show an increase in the irregular or parallelepiped agglomerates by the doping of iron (III) oxide in the host matrix. The average sizes of the agglomerates have a non-uniform and random dimensional distribution. The growth of agglomerates can be attributed to the diffusion of ions along grain boundaries, as the host matrix decreases in agglomerate size are associated with a reduction in crystallite boundary mobility. The smaller grains have an effect on the absorbance spectrum.

SEM images (Figure 9e) show that by doping, a shift in the pore size distribution to smaller values during the grain growth can be observed, which is favorable for densification. The diffusion of the iron ions corresponds to the crystallite boundary mobility.

The presence of micropores in the host matrix produces a decreasing trend in the absorption spectrum in the visible region. The microstructural modifications of the host network by doping and the presence of new crystalline phases are related to the quenching effect of the photoluminescence.

The adding of Fe_2_O_3_ to the ZrO_2_–MgO matrix results in a clear enhancement of absorbance, a decline of a photoluminescence and a change in the ceramic microstructure.

### 3.8. The Role of Nanofluids in Enhancing Thermal Efficiency and the Design of a Photothermal System Based on Ferrite-Doped Zirconia

Solar water heating systems utilize solar radiation energy to heat the working fluids, whose temperature increases, allowing this energy to be used for domestic purposes. In indirect systems, heat transfer occurs by natural convection, with thermal energy being transferred from the solar collector to the working fluid and subsequently to the domestic circuit through a heat exchanger. The low thermal conductivity of conventional working fluids limits the heat absorption and reduces heat transfer efficiency [34]. The most-used fluids are water and ethylene glycol, with thermal conductivities of 0.608 W·m^−1^·K^−1^ and 0.257 W·m^−1^·K^−1^, respectively [35]. Therefore, the thermophysical properties of the working fluid play a crucial role in heat transfer efficiency. The thermal conductivity of the base fluid can be enhanced by dispersing metallic or metal oxide nanoparticles with high thermal conductivity, leading to an increased heat transfer capability of the fluid.

The suspension of solid particles in fluids enhances the effective thermal conductivity and heat transfer efficiency, enabling a reduction in the size of heat exchange systems. Initially, the use of micron-sized suspended particles led to issues such as sedimentation, channel clogging, and equipment abrasion. In this context, nanofluids—fluids containing nanoparticles with sizes below 100 nm—were developed as a class of engineered fluids with enhanced thermal conductivity [36].

Studies have shown that the addition of small amounts of nanoparticles can significantly enhance the thermal conductivity of a fluid. For example, a copper-based nanofluid containing 10 nm particles exhibited a greater increase in thermal conductivity than an aluminum oxide nanofluid with 35 nm particles [37]. The thermal conductivity of ethylene glycol increased by 40% after the addition of 0.3 vol.% copper nanoparticles with an average diameter below 10 nm.

The proposal to enhance the thermal properties of fluids through the use of nanoparticles was introduced by Choi in 1995 [36], and since then, research has focused on the development of nanofluids with superior thermophysical properties, including thermal conductivity, thermal diffusivity, and viscosity [36]. Various types of nanoparticles have been investigated, including metals (Au, Ag, Cu, Al, and Fe), metal oxides (Al_2_O_3_, CuO, Fe_3_O_4_, SiO_2_, TiO_2_, and ZnO), carbides (SiC, TiC), and carbon-based materials (diamond, graphite, and carbon nanotubes) dispersed in base fluids such as water, water/ethylene glycol mixtures, ethylene glycol, and oils.

Table 2 presents a selection of nanofluids with low nanoparticle concentrations and the corresponding increases in thermal conductivity, demonstrating the potential of nanofluids to enhance heat transfer efficiency. For example, 0.3 vol.% copper nanoparticles dispersed in ethylene glycol increased the thermal conductivity by 40%, while 5 vol.% CuO nanoparticles in water resulted in an increase of approximately 60%.

However, not all studies report consistent enhancements in thermal conductivity. For example, an iron/ethylene glycol-based nanofluid containing 0.55 vol.% Fe nanoparticles exhibited an increase of 18%, whereas copper-based nanofluids showed increases of only 2% and 14% for nanoparticle concentrations of 0.1 vol.% and 0.55 vol.%, respectively [37]. These results suggest that Fe-based nanofluids may exhibit higher heat transfer efficiency than Cu-based ones, even though the intrinsic thermal conductivity of Fe crystals (80 W·m^−1^·K^−1^) is significantly lower than that of Cu (398 W·m^−1^·K^−1^).

Nanofluids containing ceramic nanoparticles generally exhibit more modest enhancements in thermal conductivity compared to metallic nanofluids. For instance, the addition of 1 vol.% Al_2_O_3_ nanoparticles resulted in an increase of only 4%, whereas the same volume fraction of TiO_2_ nanoparticles led to an increase of 14.4%, despite the fact that the thermal conductivity of TiO_2_ crystals (8.37 W·m^−1^·K^−1^) is lower than that of Al_2_O_3_ (77 W·m^−1^·K^−1^). The highest reported enhancement, exceeding 100%, was achieved using carbon nanotubes as nanoparticles. These results highlight the growing interest of the scientific community in nanofluids tailored for photothermal systems.

**Figure 10:**
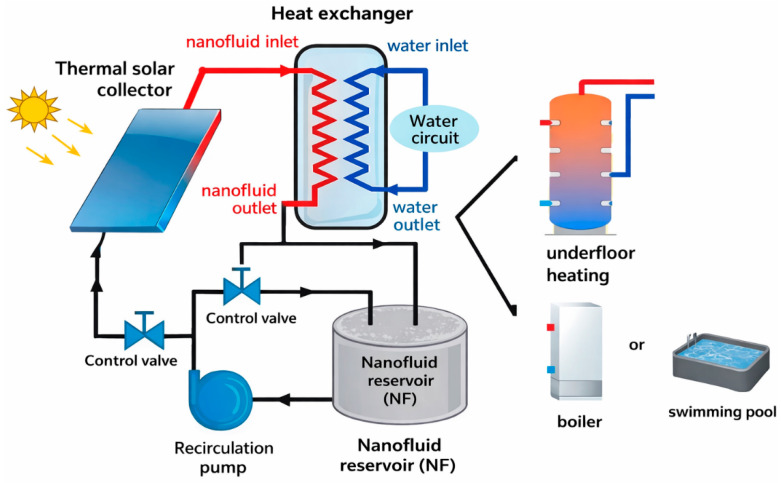
Functional schematic of a photothermal system incorporating a nanofluid circuit. Adapted from Ref. [38].

A photothermal system that employs Fe_2_O_3_-doped ZrO_2_ ceramics is proposed, combining two absorbing components within a single material, zirconia and ferrite, by ensuring efficient solar absorption and enhanced thermal conductivity. Modern photothermal systems utilize nanofluids to improve thermal efficiency, and the integration of a nanofluid circuit into an existing panel enables a reduction in the amount of absorbing material while maintaining low production costs through the simple replacement of the nanofluid (see Figure 10).

Photothermal systems incorporating a nanofluid circuit represent an advanced generation of solar thermal panels. The use of Fe_2_O_3_-doped ZrO_2_ ceramics as a nanofluid introduces a novel approach, enabling the development of innovative photothermal panels that are currently absent from the commercial market. This concept combines the functional properties of ceramic absorbers with the enhanced heat transfer performance of nanofluids. The nanofluid can be produced by the dispersion of the ceramic powder in water, followed by an ultrasonication process to ensure stable suspension [39].

In conclusion, the enhancement of the thermal conductivity is discussed only based on literature data. The development of a nanofluid and investigations regarding thermal conductivity of these materials represent a perspective for future research.

## 4. Conclusions

This paper presents the development and characterization of a new ceramic product based on black zirconia doped with ferrite, intended for use as a solar receiver in photothermal systems or as an active component in nanofluids. The structure and properties of the obtained ferrite-doped zirconia ceramic were investigated using X-Ray diffraction (XRD), UV–Vis spectroscopy, electron spin resonance (ESR), scanning electron microscopy (SEM), and optical band gap energy determination.

- XRD analysis revealed the presence of monoclinic and tetragonal zirconia crystalline phases, as well as of crystalline phases of ferrite and magnesium oxide. The average particle size was below 90 nm.
- SEM micrographs showed homogeneous regions with well-defined grains.
- UV–Vis spectra indicated high absorption in the ultraviolet, visible, and infrared regions, with an absorption of about 80% and a less than 20% reflectance in the visible range.
- The optical band gap energy values confirmed the semiconducting nature of the obtained ceramic.
- Ferrite doping significantly improves the optical properties of zirconia compared to the undoped (white) zirconia, resulting in the recommendation of this material for solar absorber applications in photothermal panels.
- EPR spectra revealed resonance lines characteristic of Fe^3+^ ions.
- The implementation of a nanofluid circuit for future applications can reduce the amount of the required absorbing material and will enable the development of new innovative photothermal panels that are currently available on the market.
- This study demonstrates the feasibility of using ferrite-doped zirconia as an efficient and versatile solar receiver material.
- The obtained results provide a solid foundation for the development of next generation photothermal systems with enhanced absorption and reduced costs.

## Figures and Tables

**Table 1:** Lattice parameters (*a*, *b* and *c*) and effective crystallite mean size (*D_eff_*) of iron–zirconia ceramics.

Crystalline Phase	Proportion [%vol]	*a* [Å]	*b* [Å]	*c* [Å]	β [Degrees]	*D_eff_* [nm]
MgFe_2_O_4_	28.2	8.3947	8.3947	8.3947		66.88
*m*—ZrO_2_	65	5.1516	5.2020	5.3129	99.077	80.87
t—ZrO_2_	6.8	3.6083	3.6083	5.1999		27.01
MgO	6.6	4.1893	4.1893	4.1893		9.37
t—ZrO_2_	5.4	3.6071	3.6071	5.2114		26.21
*m*—ZrO_2_	44.4	5.1487	5.2016	5.3117	99.058	88.2
Fe_3_O_4_	43.5	8.3953	8.3953	8.3953		73.9

**Table 2:** Thermal conductivity reported for different nanofluids [37,38]. The nanofluids were prepared by the dispersing of the nanoparticles in the working fluid and ultrasonic treatment.

Nanoparticle	Particle Size (nm)	Working Fluid	Nanoparticle Concentrations (vol.%)	Thermal Conductivity Enhancement (%)
Ag	<100	Water	0.3–0.9	from 30 la 50 °C
Ag	100–500	Ethylene glycol	0.1–1.0	18
Cu	50–100	Apă	0.1	24
Cu	<10	Ethylene glycol	0.01–0.05	41
Fe	10	Ethylene glycol	0.1–0.55	18
Al_2_O_3_	650–1000	Oil	0.5–4	20
CuO	100	Water	7.5	52
SiO_2_	12	Ethylene glycol	1–4	23
TiO_2_	15	Water	0.5–5	30
Carbon	190	Water	4.4–7.7	from 10 to 35 °C
Carbon nanotubes	25 nm × 50 µm, diameter 10 nm	Oil	1	150

## Data Availability

The original contributions presented in this study are included in the article/Supplementary Materials. Further inquiries can be directed to the corresponding authors.

## Supplementary Materials

The following supporting information can be downloaded at: https://www.mdpi.com/article/10.3390/nano16060346/s1, Figure S1: The Fe_3_O_4_ or MgFe_2_O_4_ phase identification from X-ray powder diffraction data using MATCH! software for the ZrO_2_-MgO-Fe_2_O_3_ sample; Table S1: Some properties of the MgFe_2_O_4_ and Fe_3_O_4_.
