# Supplementary material for: Zirconia Ceramics Doped with Ferrite for Solar Thermal Systems
Source: Nanomaterials (Basel). 2026 Mar 11;16(6):346. doi: 10.3390/nano16060346 (PMC13029176; doi:10.3390/nano16060346)
Supplement: Supplementary file 1 [file nanomaterials-16-00346-s001.zip › nanomaterials-4181367-supplementary.pdf]

The reflection of diffraction peaks of the Fe_3O_4 crystalline phase is overlapped with $MgFe_2O_3$ crystalline phase in X – ray diffractogram (see *Figure S1*).

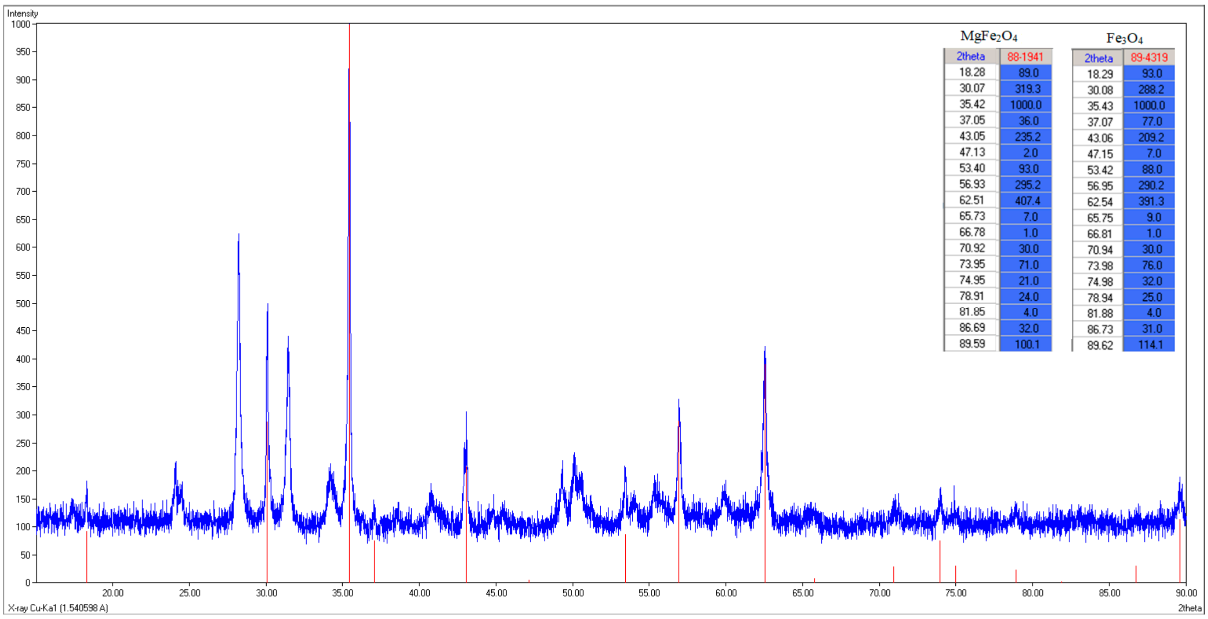

Figure S1: The Fe_3O_4 or $MgFe_2O_4$ phase identification from X – ray powder diffraction data using MATCH! software for the ZrO_2 – MgO – Fe_2O_3 sample.

The summary comparison between $MgFe_2O_4$ and Fe_3O_4 crystalline phase are shown in *Table S1*.

The values of the standard enthalpy of formation (at 298K) are approximatively – 1400 KJ / mol for magnesium ferrite and – 1120 KJ / mol for Fe_3O_4. $MgFe_2O_4$ is thermodynamically more stable than Fe_3O_4.

Both crystalline phases are inverse spinel ferrites and their UV – Vis and PL spectra share similarities. Differences in cation distribution (Mg^{2+} versus Fe^{2+}/Fe^{3+}) cause measurable spectral differences.

$MgFe_2O_4$ shows a defined band gap edge at ~ 1.8 - 2.2 eV, clear absorption in UV – Vis spectra and intense PL band while Fe_3O_4 shows broad, nearly metallic absorption without a sharp edge and PL bands. In our ceramic the values of the gap energy for direct and indirect transitions are situated between 1.81 – 1.96 eV, shows a clear absorption edge in UV – Vis spectra and

stronger PL intensity. Fe_3O_4 shows strong broad UV – Vis absorption across visible region whereas $MgFe_2O_4$ indicates absorption edge in UV - Vis.

PL intensities in ferrites are originated from Fe^{+3} d – d transitions, oxygen vacancy defects and surface states. For Fe_3O_4 are very weak PL and fast nonradioactive recombination. At $MgFe_2O_4$ the PL intensity are stronger than that Fe_3O_4 and have better radiative recombination.

Table S1. Some properties of the $MgFe_2O_4$ and Fe_3O_4.

**Property**	**$MgFe_2O_4$**	**Fe_3O_4**
Enthalpy of formation (ΔH_f)	~ - 1400 KJ/mol	~ -1120 KJ/mol
Band gap energy, E_g	~ 1.8 – 2.2 eV	~ 0.1 – 0.3 eV
UV - Vis	Clear absorption edge	Broad metallic absorption
PL intensity	Moderate	Very weak or almost no PL
Electrical behavior	Semiconductor	Semimetal / narrow - gap
Line shape in EPR<br><br>g - value	Broad resonance line but symmetric<br>$g \approx 2.0 – 2.22$<br>sometimes weak signal near $g \approx 4.3$ (isolated Fe^{3+})	Very large and asymmetric<br><br>$g \approx 2.1 – 2.3$ (broadened)

Line shape in the EPR spectrum is broad and symmetric with g value situated between 2 and 2.22 for $MgFe_2O_4$. The EPR signal is also very large, often asymmetric and with intense super-exchange interactions at Fe_3O_4. In iron ceramic the linewidth of EPR signal is moderate, symmetric, with g ~ 2.0 – 2.22 and with intense dipole – dipol interactions. EPR alone at room temperature strongly suggests $MgFe_2O_4$ rather than Fe_3O_4 in our spectrum.

From comparative studies of the varied properties we can conclude the presence of $MgFe_2O_4$ crystalline phase in the Fe_2O_3 – MgO – ZrO_2 ceramic.
